# Adaptation to Endophytic Lifestyle Through Genome Reduction by *Kitasatospora* sp. SUK42

**DOI:** 10.3389/fbioe.2021.740722

**Published:** 2021-10-12

**Authors:** Noraziah M. Zin, Aishah Ismail, David R. Mark, Gareth Westrop, Jana K. Schniete, Paul R. Herron

**Affiliations:** ^1^ School of Diagnostic and Applied Health Sciences, Universiti Kebangsaan Malaysia, Kuala Lumpur, Malaysia; ^2^ Strathclyde Institute of Pharmacy and Biomedical Sciences, University of Strathclyde, Glasgow, United Kingdom

**Keywords:** endophyte, *Kitasatospora*, genome, pan-genome, Antidesma neurocarpum miq

## Abstract

Endophytic actinobacteria offer great potential as a source of novel bioactive compounds. In order to investigate the potential for the production of secondary metabolites by endophytes, we recovered a filamentous microorgansism from the tree *Antidesma neurocarpum* Miq. After phenotypic analysis and whole genome sequencing we demonstrated that this organism, SUK42 was a member of the actinobacterial genus *Kitasatospora*. This strain has a small genome in comparison with other type strains of this genus and has lost metabolic pathways associated with Stress Response, Nitrogen Metabolism and Secondary Metabolism. Despite this SUK42 can grow well in a laboratory environment and encodes a core genome that is consistent with other members of the genus. Finally, in contrast to other members of *Kitasatospora*, SUK42 encodes saccharide secondary metabolite biosynthetic gene clusters, one of which with similarity to the acarviostatin cluster, the product of which displays α-amylase inhibitory activity. As extracts of the host plant demonstrate this inhibitory activity, it suggests that the potential medicinal properties of *A. neurocarpum* Miq might be provided by the endophytic partner and illustrate the potential for exploitation of endophytes for clinical or industrial uses.

## Introduction

Natural products hold great potential for novel drug discovery and members of the actinobacteria are amongst the most prolific producers of bioactive natural compounds (Barka et al., 2016). Despite their abundance in soil, more recently alternative environments such as plant tissues have been investigated for their discovery (Barka et al., 2016). Such organisms include symbiotic, endophytic actinobacteria that occupy an ecological niche within plant tissues and have recently begun to generate great interest as a potential source of novel natural products with industrial, environmental or clinical applications (Zin et al., 2007; Singh and Dubey, 2018). Endophytes enjoy mutualistic or antagonistic, but not parasitic, relationships with their host and can have important effects on plant growth through the production of hormones or other growth-influencing factors. They are also able to enhance increase resistance to abiotic and biotic stresses, as well as providing resistance to pests and pathogens. In exchange, the host plant offers food and shelter for these symbiotic organisms (Singh and Dubey, 2018). Endophytic actinobacteria, especially from the genus *Streptomyces*, are known to synthesize active compounds which make these bacteria a rich source of natural products particularly for clinical applications (Strobel, 2003), such as antimalarial (Ahmad et al., 2021) or antibacterial agents (Alshaibani et al., 2017). For example, the endophytic strain *Streptomyces* SUK25 can produce various diketopiperazine derivatives, with bioactivity against pathogenic bacteria such as MRSA and *Enterococcus raffinosus* whilst possessing low toxicity against HepaRG cells (Alshaibani et al., 2017) and illustrates the rich prospects offered by drug discovery from endophytes.

The family *Streptomycetaceae* includes the genera *Streptomyces* and *Streptacidiphilus* (Nouioui et al., 2018)*,* as well as *Kitasatospora* (Omura et al., 1982; Takahashi, 2017). Like *Streptomyces*, members of the genus *Kitasatospora* are known to produce active compounds such as setamycin (bafilomycin B1) and bafilomycin A1, a proteasome inhibitor and an anti-fungal agent (Momose et al., 2001; Yoon et al., 2006; Ichikawa et al., 2010). Members of the *Kitasatospora* are non-fastidious in their nutritional requirements and form leathery colonies with both vegetative and aerial hyphae. The latter develop further to form 20 or more spores per chain. As such, their development closely resembles that of members of *Streptomyces* (Takahashi, 2017). Although once a subject of debate (Wellington et al., 1992), the classification of the *Kitasatospora* has been resolved by the application of 16S rRNA sequencing (Labeda et al., 2012) and whole genome sequencing (Nouioui et al., 2018; Li et al., 2021). The genus *Kitasatospora* is also distinguishable from *Streptomyces* on the basis of cell wall composition (Ichikawa et al., 2010), although more recently, another criterion that can be used to distinguish between these two genera is through the use of the SsgB amino acid sequence (Keijser et al., 2003; Willemse et al., 2011). SsgB is a member of the SALPs (SsgA-like proteins), which are present in all members of the *Streptomyceteaceae*. SALPs play an important role in the control of cell division and morphogenesis in actinobacteria with complex life cycles (Traag and van Wezel, 2008). SsgB recruits the cytokinetic protein FtsZ, which is responsible in initiating sporulation-specific cell division in an SsgA-dependent manner (Willemse et al., 2011). Importantly, SsgB displays conservation within a single genus while between genera variation is as low as 40–50% (Girard et al., 2013). This makes SsgB ideal as a tool for molecular systematic differentiation *of Streptomyces* and *Kitasatospora*.

Earlier work reported the isolation and classification *of Streptomyces kebangsaanensis* sp. from a Malaysian ethnomedicinal plant (Sarmin et al., 2013). In order to investigate genome mining for natural product discovery from endophytic bacteria from indigenous Malaysian plants, we isolated one organism, SUK42, from the plant *Antidesma neurocarpum* Miq with reported α-glucosidase inhibitory activity (Elya et al., 2012). This strain was initially believed to be a member of the *Streptomyces*, however further analysis revealed that this strain was a member of the rare genus *Kitasatopsora*. As such, we decided to confirm this initial identification, carry out comparative genomics with other members of this genus and explore the strains potential for production of secondary metabolites through genome mining.

## Methods

### Isolation and Growth Conditions

SUK42 was isolated from the internal tissue of stem from the plant, *Antidesma neurocarpum* Miq, collected at the UKM forest reserve Bangi, Malaysia. The plant sample was first subjected to surface sterilisation (Coombs and Franco, 2003) in order to remove epiphytic organisms. The outer layer of plant stem was removed and the inner tissue was excised and plated on water agar (Coombs and Franco, 2003), pH 7.2 and supplemented with cycloheximide (50 μg ml^−1^) and nystatin (50 μg ml^−1^) before incubation at 27°C. SUK42 was purified and maintained on ISP2- agar and stored using 20% (v/v) glycerol at −80°C.

### Phenotypic Characterisation

Production of melanin was examined on ISP-7 agar after 14 days incubation at 30°C. Agar without inoculation was used as a control and production of a dark brown to black pigmentation produce recorded as positive. Tolerance to NaCl (1–15%), pH (3–12) and temperature (10, 30, 37, and 50°C) were analyzed according to (Gordon et al., 1974) on modified ISP-2 agar incubated for 14 days at 30°C. Degradation of adenine, hypoxanthine, L-tyrosine, and xanthin was analysed according to Gordon et al. (1974). Carbon source utilization growth on the isomeric form of diaminopimelic acid was determined by growth on ISP-9 agar supplemented with 1% (w/v) carbon sources (Shirling and Gottlieb, 1966) after incubation at 30°C. Hydrolysis of aesculin and starch and decomposition of casein and urea was carried out using established procedures (Gordon et al., 1974) and nitrate reduction determined by inoculation into nitrate broth supplemented with sulphanilic acid (Gottlieb, 1961).

Cell wall analysis were done following growth on ISP-2 agar at 28°C for 14 days. Cells were harvested by centrifugation at 12,000 × g, washed twice with sterile dH_2_O and dried before dissolving in 1 ml 6N HCL as described previously (Staneck and Roberts, 1974). Standard solutions of DAP (DL-diamonipimelic acid) and LL-DAP were and thin layer chromatography plates developed with ninhydrin solution before drying at 80°C) (Staneck and Roberts, 1974).

### Genomic DNA Extraction, Sequencing and Assembly

Genomic DNA from SUK42 was obtained from cultivation of SUK42 in ISP-2, extracted according to established procedures and 16S rRNA sequence amplified using universal bacterial 16S rRNA gene primers (Coombs and Franco, 2003). PCR products were sequenced and compared to existing sequences using EzBioCloud (Yoon et al., 2017). Whole genome sequencing of strain SUK42 were done using an Illumina MiSeq system (first base Company) with 301 bp paired-end reads. The sequence was assembled using Unicycler version 0.4.8 (Wick et al., 2017) and assembly quality assessed using Quast (Mikheenko et al., 2018) and viewed in CG viewer (Grant and Stothard, 2008).

### Bioinformatic Analysis

To place SUK42 in its phylogenetic context, related strains were identified using AutoMLST with SUK42 as the query sequence (Alanjary et al., 2019) after a concatenated alignment. Reference sequences of type strains (Supplementary Table S1) were identified using AutoMLST and used to scaffold the assembly of SUK42 with the other reference sequences with Medusa (Bosi et al., 2015). Average nucleotide identity values were determined using OrthoANI (Lee et al., 2016) and alignments of SsgB amino acid sequences were carried using Mega X (Kumar et al., 2018). Pan-genome analysis was carried using Roary version 3.13.0 following annotation by Prokka, version 1.14.6 (Seemann, 2014), available on Galaxy (Afgan et al., 2016). Outputs from Roary were displayed in Phandango (Hadfield et al., 2018). Collections of functional roles that make up metabolic pathways were identified as subsytems established by SEED-viewer analysis of genome sequences by RASTtk (Brettin et al., 2015). Secondary metabolite biosynthetic gene clusters were first identified by subjecting genome sequences to antiSMASH, Galaxy ver. 5.1.2, (Blin et al., 2019), outputs from antiSMASH were then further investigated using BiG-SCAPE (Navarro-Munoz et al., 2020) to sort clusters into higher order gene cluster families. Chord diagrams were plotted using the circlize package in R (Gu et al., 2014).

## Results

### Phenotypic Characterisation of SUK42

**FIGURE 1:**
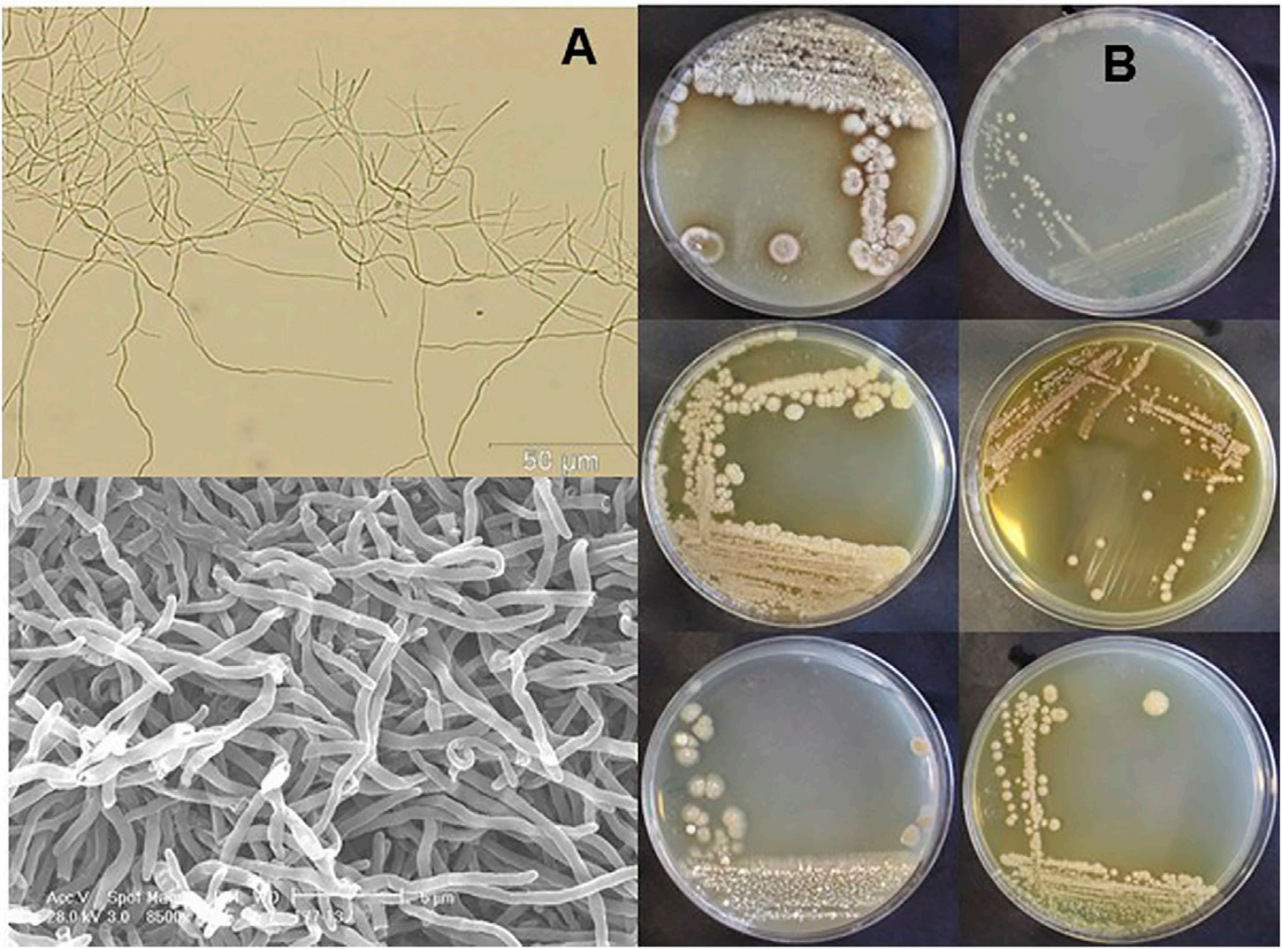
Mycelial morphology **(A)** and macromorphology **(B)** of *Kitasatospora* sp. SUK42. **(A)** Scanning electron **(top)** and bright field microscope images **(bottom)** of ISP2-grown *Kitasatospora* sp. SUK42 substrate mycelium. **(B)** Plates of *Kitasatospora* sp. SUK42 gown on ISP-2 **(top)**, ISP-3, ISP-4 **(left hand column)** and ISP-4 **(top)**, ISP-5, ISP6 **(right hand column)**.

In order to identify novel endophytic actinobacteria, SUK 42 was isolated from the internal tissue of stem from the plant, *Antidesma neurocarpum* Miq. 16S rRNA sequencing indicated that SUK42 was most closely related to *Streptomyces xanthocidicus* (data not shown). This strain has now been renamed *Kitasaotospora xanthocidicus* (Nouioui et al., 2018). Following cultivation, macro-morphological characterization was carried out following standard protocols (Shirling and Gottlieb, 1966), aerial mycelia were observed on ISP-3 and ISP-4 whilst pigmentation was observed on all agars (Figure 1B); melanin was not produced on ISP-7 (data not shown). Microscopic observation of mycelial growth on ISP-2 was made by light microscopy and scanning electron microscopy. (Figure 1A). SUK42 could grow at temperatures up to 50°C, a pH of 12 and 10% w/v NaCl and could decompose both starch and casein. Dextrose, galactose, sucrose, maltose, glucose, rhamnose, and sorbitol were used as sole carbon, but inositol and D-mannitol were not. Hydrolysis of l-tyrosine and starch were observed, but reduction of nitrate and decomposition of urea were not. Both L,L and meso-diaminopimelic acid were detected in SUK42 when grown on ISP-2 media (data not shown).

### Genomic Characterisation of SUK42

Genome sequencing of SUK42 was carried out using whole-genome sequencing by 1st Base Malaysia performed on an Illumina Miseq apparatus. We obtained 51.6 million reads that, following assembly, were assembled into 35 contigs of >200 bp. The total size of the assembly was 6,568,264 with a G + C content of 72.9%. The sequence is deposited with NCBI under accession number LFMD00000000 and assembly GCA_015776785.2. Automatic functional annotation results were obtained using the NCBI Prokaryotic Genome Annotation Pipeline (http://www.ncbi.nlm.nih.gov/genomes/static/Pipeline.html) and a genomic map is displayed in Supplementary Figure S1. SUK42 has a relatively small genome of 6.5 Mb compared to other strains of *Kitasatopora* [mean size 8.7 Mb (Li et al., 2021)].

### SUK42 is a Member of the Genus *Kitasatospora*

**FIGURE 2:**
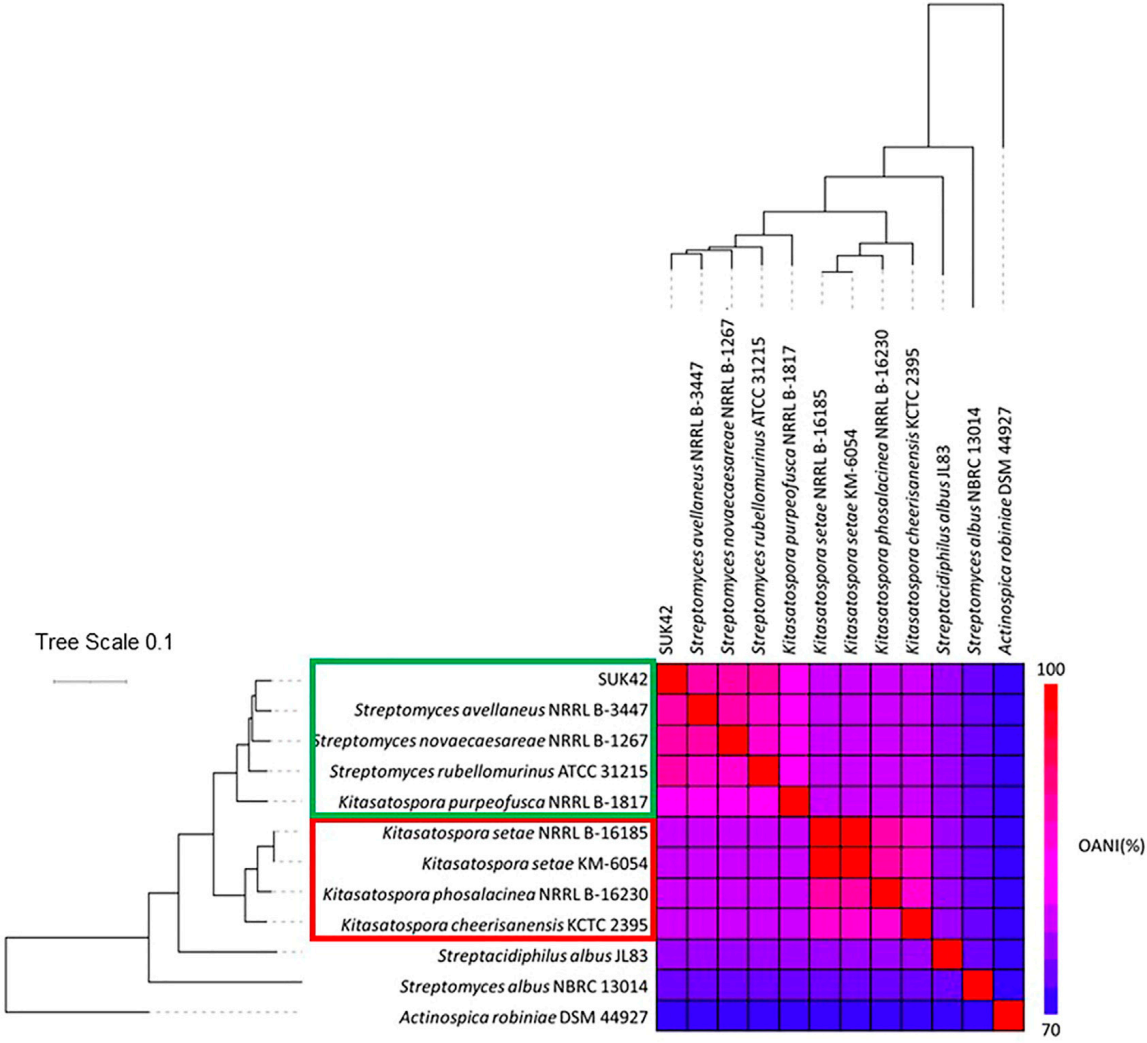
Phylogenetic relationship of putative members of the genus *Kitasatospora.* Eight strains with >84.7% ANI with Kitasatospora sp. SUK42 and appropriate type were used to carry out Multi-Locus Sequence Analysis (MLSA) to produce a high-resolution species tree using AutoMLST after a concatenated alignment (Alanjary et al., 2019). All branches were supported with boot strap values of 100 except the *Kitasatospora* sp. SUK42/S*. avellaneus* node shared with *S. novoceasaereae* (82). The average nucleotide identities (ANI) was calculated using the OrthoANI tool (Lee et al., 2016) and plotted with the heat map function in R (Ver. 4.0). *Actinospica robiniae*, type strain of the family Actinospicaceae and the type strains of the genera *Streptomyces* and *Streptacidophilus*, *S. albus* NBRC 13014 and *S. albus* JL83, respectively, were used as outgroups.

In order to identify SUK42 more accurately, we analysed the assembled sequence using AutoMLST (Alanjary et al., 2019). This analysis identified eight type strains available as reference sequences that had an estimated ANI of >84.7% with SUK42 (listed in Supplementary Table S1). The next most similar type strains were members of the genus *Streptacidiphilus* (*Streptacidiphilulus rugosus* AM-16 had an ANI <81.6% compared to SUK42). The eight type strains were named as both members of the genera *Streptomyces* and *Kitasatospora*, so in order to better delineate these organisms, we subjected their genome sequences to further analysis of these strains using OrthANI (Figure 2) along with type stains of neighbouring genera (*Actinospica robiniae* DSM44927, *Streptacidiphilus albus* JL83, *Streptomyces albus* NBRC 13014). This demonstrated that SUK42 was most closely related to *Streptomyces avellaneus* of the eight type strains that were cleary distinct form the out-groups from neighbouring genera. However the presence of members of the *Kitasatospora* in this clade suggested that *Streptomyces avellaneus*, *Streptomyces novoceasareae*, and *Streptomyces rubellomurinus* might have been misclassified. Subsequently the mis-classification of these strains was confirmed in the literature (Li et al., 2021). It was noticeable that the type strains of the genera *Streptomyces* (*Streptomyces albus* NBRC 13014) and *Streptacidiphilus* (*Streptacidiphilus albus* JL83) were phylogenetically separate from SUK42 and the other eight strains (Figure 2) and confirms the grouping of SUK42 with members of *Kitasatspora* and distinct from the other two genera that form members of the family Streptomycetaceae. Interestingly, there were two sub-clades contained with the collection of the *Kitasatospora* type strains (Group 1: *Kitasatospora* Sp. SUK42, *S. avellaneus* NRRL B-3447, *S. novaecaesareae* NRRL B-1267, *S. rubellomurinus* ATCC 31215, *K. purpeofusca* NRRL B-1817 and Group 2: *K. setae* NRRL B-16185, *K. setae KM-6054*, *K. phosalacinea* NRRL B-16230, *K. cheerisanensis* KCTC 2395).

**FIGURE 3:**
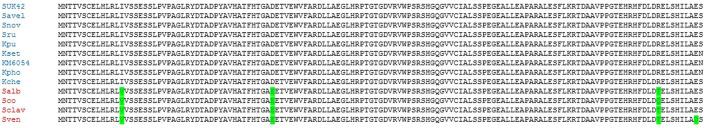
Alignment of SsgB sequences from *Kitasatospora* sp. SUK42, other putative *Kitasatospora* reference sequences and model strains from the genus *Streptomyces*. SsgB sequences were aligned using Clustal Omega in Mega (Kumar et al., 2018). Amino acid substitutions in model strains of S*treptomyces* are highlighted in green.

To confirm the phylogenetic position of SUK42 amongst the genus *Kitasatospora*, we extracted and analysed the amino acid sequences of the cell division protein SsgB (Girard et al., 2013) from our collection of Kitasatospora sequences in addition to the model members of the genus *Streptomyces* (*S. albus*, *S. coelicolor*, *S. clavuligerus*, and *S. venezuelae*). SsgB is responsible for FtsZ ring placement in streptomycetes (Keijser et al., 2003; Willemse et al., 2011). A comparison of SsgB sequences from the two genera defined three amino substitutions (V14-I14; E46-D46 and T/Q/K128–R46l *Streptomyces*-*Kitasatospora,* respectively). As shown in Figure 3, SUK42 and type other type strains show the substitutions diagnostic of *Kitasatospora* and demonstrate that both these organisms are indeed members of this genus. Consequently, our studies of the SsgB amino acid sequence indicate that, like SUK42, *S. avellaneus, S. novoceasareae,* and *S. rubellomurinus* should be classified as members of the genus *Kitasatospora*.

### Pan-Genome of *Kitasatospora*

**FIGURE 4:**
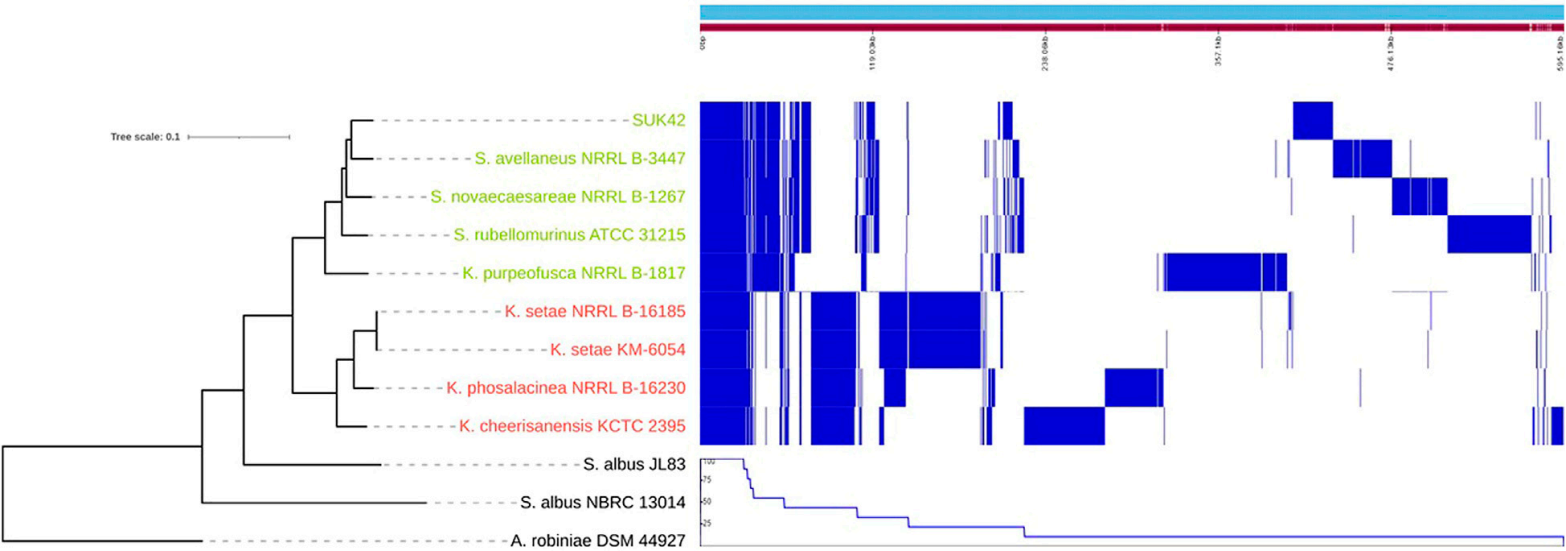
Core and pan genome of *Kitasatospora* sp. SUK42 and other putative *Kitasatospora* reference sequences. Phylogenetic tree, core, shell, cloud and pan-genomes of *Kitasatospora* sp. SUK42 and the other eight putative *Kitasatospora* reference sequences (Supplementary Table S1) were determined using Roary (Page et al., 2015) and displayed using Phandango (Hadfield et al., 2018). Group 1 *Kitasatospora* strains are shown in green and Group 2 in red.

Due to the relatively small genome size of SUK42, we wanted to investigate if any specialisation to an endophytic lifestyle existed. To understand which genes were unique (accessory genome) or conserved (core) genome (Page et al., 2015) among the *Kitasatospora* reference sequences a comparative pan-genome analysis was performed using whole-genome sequences. The pan-genome of the eight reference sequences (Supplementary Table S1)and *Kitasatopsora* sp. SUK42 was evaluated using Roary set at minimum percentage identity for blastp of 80% (Corretto et al., 2017) (Table 1) to cluster the genes encoding complete protein sequences into core (hard core and soft core) and accessory (shell and cloud) genomes. It was possible to identify 29,756 gene families comprising the pan-genome and of those, 1,498 (∼5%) were present in the core genome core (>95% of strains), and the remaining 28,528(∼95%) as accessory (present <95% of strains) (Table 1). In a recent study of all available *Kitasatospora* sequences, but not SUK42 (Li et al., 2021), the core genome of this genus was estimated to be 1,476 gene families and the similarity of these two values suggests that SUK42 retains the essential components of the *Kitasatospora* core genome. When examined in a phylogenetic context, the pan-genome obtained with Roary allowed us to determine the presence or absence of genes in each genome and also to compare and identify core regions from the two *Kitasatopsora* groups (Figure 4). Once more, phylogenetic analysis showed that isolates within each group affiliated closely with each other but not with strains from the other group. In this way, strains from Group 1 (green) clustered closely with *Kitasatospora* sp. SUK42 and were distinct from Group 2. However, a core genome was observed containing shared genes between both groups. Furthermore, genes only present within one group were identified creating two distinct regions that are part of a group-specific core genome.

**TABLE 1:** Core and pan genome sizes of *Kitasatospora* sp. SUK42 and other putative *Kitasatospora* reference sequences Summary of the number of genes comprising the core, shell, cloud and pan-genomes of *Kitasatospora* sp. SUK42 and other eight putative *Kitasatospora* reference sequences (Supplementary Table S1) were determined using Roary (Page et al., 2015).

Core genes	(99% ≤ strains ≤100%)	1,498
Soft core genes	(95% ≤ strains <99%)	0
Shell genes	(15% ≤ strains <95%)	9,664
Cloud genes	(0% ≤ strains <15%)	18,594
Total genes	(0% ≤ strains ≤ 100%)	29,756

### Subsystems Involved in Carbohydrate and Amino Acid Metabolism Are Enriched in *Kitasatospora* SUK42

**FIGURE 5:**
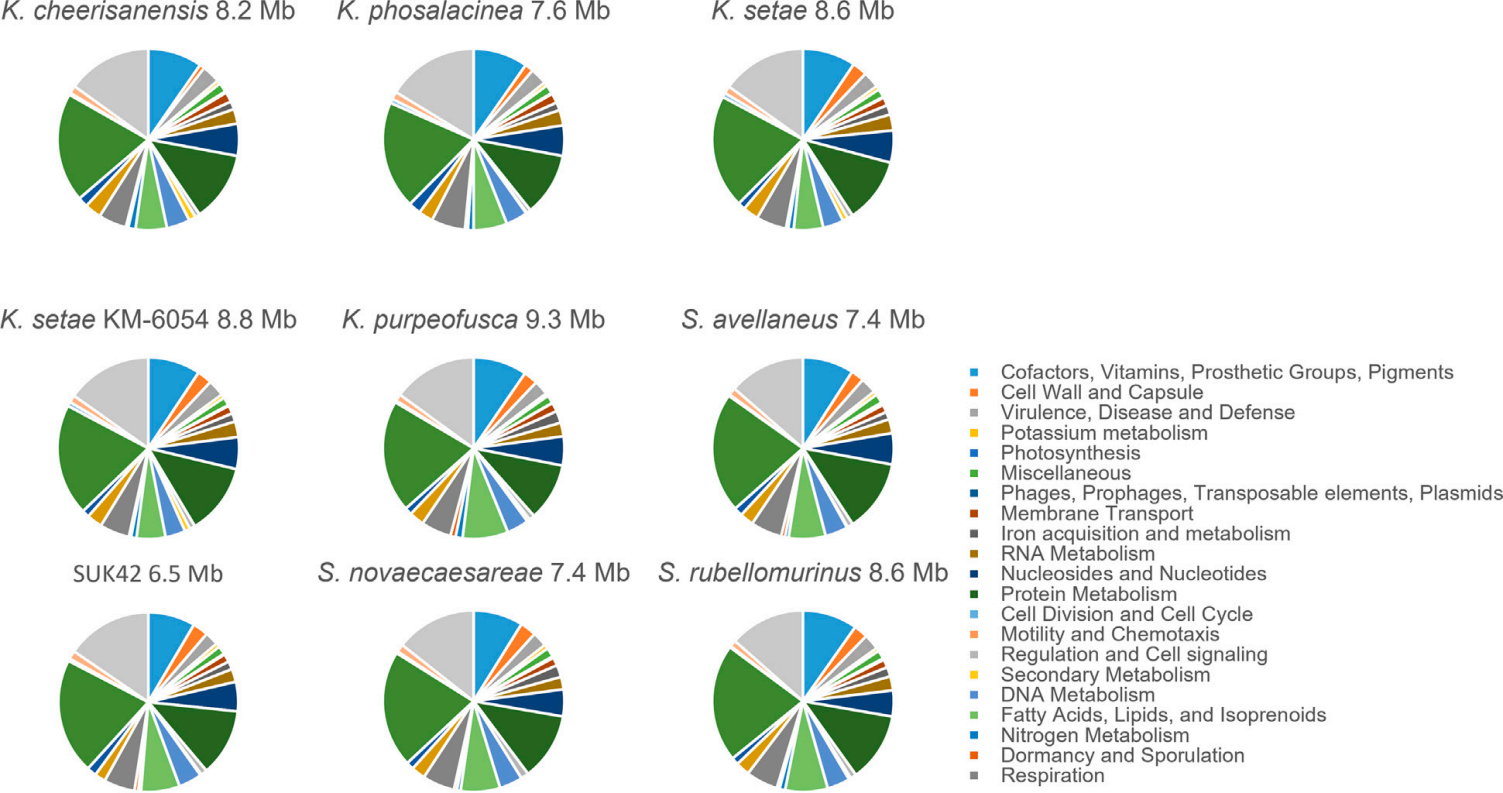
Sub-system comparison of *Kitasatospora* sp. SUK42 and other putative *Kitasatospora* reference sequences. Pie charts showing SEED sub-systems determined for *Kitasatospora* sp. SUK42 and eight other *Kitasatospora* type strains using RASTtk (Brettin et al., 2015).

In order to further investigate the functional categories of the genes encoded by *Kitasatospora* SUK42, we subjected this strain and the eight *Kitasatospora* type strains to analysis by RASTtk (Brettin et al., 2015) that provided high-quality assessments of gene functions and an initial metabolic reconstruction. Annotation data in the SEED (Overbeek et al., 2005), where genes are organized into sets of logically related functional roles (subsystems) allowed us to compare subsystems between the nine *Kitasatospora* genome sequences (Figure 5). Although the relatively small genome size of *Kitasatospora* SUK42 led us to predict that this strain would show reduced numbers (Table 2) of subsystems than the average for *Kitasatospora* reference sequences (Table 2) some subsystems were more or less represented than others. SUK42 encodes 94.25% of the average number of subsystems per *Kitasatospora* genome, however subsystems involved in Cell Wall and Capsule formation; Dormancy and Sporulation; Phosphorus Metabolism; Metabolism of Aromatic Compounds; Regulation and Cell signalling; Potassium metabolism; Fatty Acids; Lipids; and Isoprenoids; DNA Metabolism were over-represented in respect to the average (Table 2). In contrast subsystems involved with Carbohydrates; Amino Acids and Derivatives; Protein Metabolism; Respiration; Nucleosides and Nucleotides; Miscellaneous; Membrane Transport; Sulphur Metabolism; RNA Metabolism; Cofactors, Vitamins, Prosthetic Groups, Pigments; Iron acquisition and metabolism; Virulence, Disease and Defense; Phages, Prophages, Transposable elements, Plasmids; Stress Response; Nitrogen Metabolism; Secondary Metabolism were under-represented with respect to the average (Table 2). Consequently, if the endophytic life-style of SUK42, has led to its small genome size, it seems that the disproportionate loss of the latter subsystems has contributed to the reduction in genome size.

**TABLE 2:** Subsystems from SUK42 and *Kitasatospora* reference sequences. SUK42 and other *Kitasatospora* reference sequences (RS average) were analysed by RASTtk to establish collections of functional roles and were identified as subsytems by SEED-viewer analysis. The percentage of each SUK42 subsystem was calculated with respect to the RS average.

	SUK42	Rs average	% SUK42 of Rs average
Genome Size	6,548,107	8,246,635	79
Number of Subsystems	296	312	95
Number of Coding Sequences	5,887	7,654	77
Number of RNAs	70	81	86
Subsystems			
Cell Wall and Capsule	50	45	111
Dormancy and Sporulation	12	11	109
Phosphorus Metabolism	27	26	103
Metabolism of Aromatic Compounds	31	30	102
Regulation and Cell signaling	20	20	101
Potassium metabolism	13	13	100
Fatty Acids, Lipids, and Isoprenoids	125	129	97
DNA Metabolism	75	78	96
Carbohydrates	277	296	93
Amino Acids and Derivatives	374	406	92
Protein Metabolism	218	240	91
Respiration	100	110	91
Nucleosides and Nucleotides	95	107	89
Miscellaneous	27	32	86
Membrane Transport	26	31	84
Sulfur Metabolism	8	10	83
RNA Metabolism	42	52	81
Cofactors, Vitamins, Prosthetic Groups, Pigments	152	190	80
Iron acquisition and metabolism	26	34	78
Virulence, Disease, and Defense	44	58	76
Phages, Prophages, Transposable elements, Plasmids	3	4	71
Stress Response	35	53	66
Nitrogen Metabolism	9	20	46
Secondary Metabolism	4	11	36
Photosynthesis	0	0	
Cell Division and Cell Cycle	0	0	
Motility and Chemotaxis	0	0	

### Secondary Metabolism in *Kitasatospora* sp. SUK42

**FIGURE 6:**
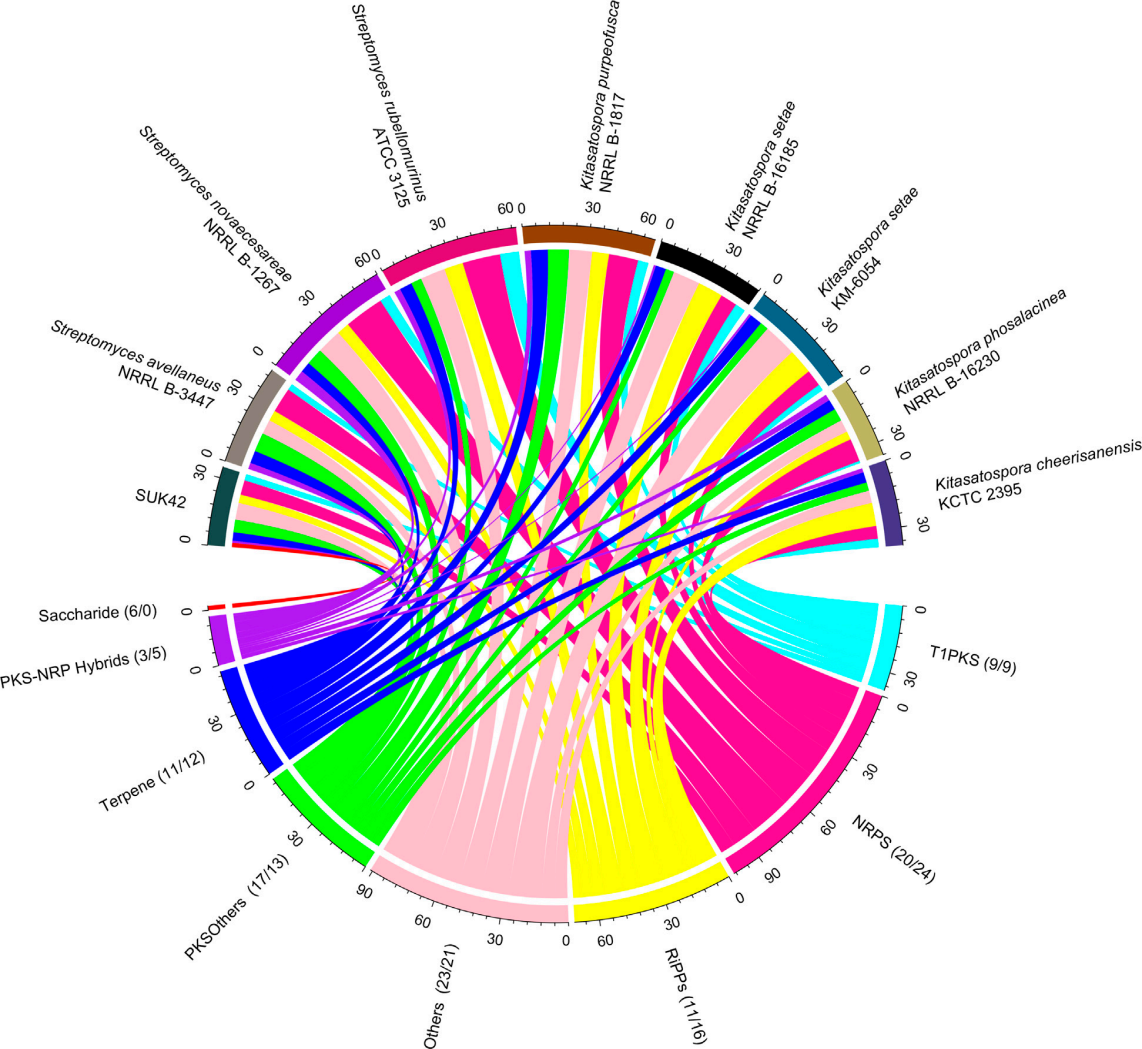
SUK42 is the only member of *Kitasatospora* reference sequences to encode Saccharide-type BGCs. Chord diagram illustrating the distribution of Gene Cluster Families amongst SUK42 and related strains. Of the eight, *Kitasatospora* sequences was the only one to encode Saccharide type clusters. Values in brackets are the percentage of clusters in a GCF that belong to SUK42 or the eight reference sequences, respectively. Gene clusters were first identified using AntiSMASH (Blin et al., 2019) before assigned to individual families using BIG-SCAPE (Navarro-Munoz et al., 2020). Plotted using the circlize package in R.

Subsystems analysis demonstrated that SUK42 contained fewer metabolic pathways associated with secondary metabolism than the average of the eight reference sequences. So, to determine the genomic potential *Kitasatospora* sp. SUK42, the eight *Kitasatospora* type strains and representatives of neighbouring genera (*Actinospica robiniae* DSM44927, *Streptacidiphilus albus* JL83, *Streptomyces albus* NBRC 13014), for secondary metabolite production in more detail, we examined their genomes using antiSMASH to predict biosynthetic gene clusters (BGCs) (Blin et al., 2019). When genomes sequences are comprised of many contigs, this can lead to misidentification of BGCs due to contig edge effects. With the exception of *K. setae* KM-6054 our collection of genome sequences consisted of more than one contig so we constructed similarity networks using BiG-SCAPE (Navarro-Munoz et al., 2020) and identified secondary metabolite biosynthetic gene cluster families (GCFs) based on Pfam similarities (Figure 6). Using antiSMASH we predict that SUK42 encodes 28 regions containing BGCs, five of which exist at contig edges (Supplementary Table S2). Of these 28 clusters, only five generated ClusterBLAST hits with similarity >75% to known BGCs. 35 GCFs were predicted using BiG-SCAPE, whilst the eight type strains encoded average of 54.125 GCFs per genome (total = 433). Of these, SUK42 encodes a smaller percentage of GCFs than the eight type strains in the following major biosynthetic classes, respectively: NRPS (20 vs. 24%), RIPPs (11 vs. 16%), Terpenes (11 vs. 12%) and PKS-NRP_Hybrids (3 vs. 5%). Conversely, a larger percentage in the GCFs in SUK42 are found in the biosynthetic categories Others (23 vs. 21%), PKSI (17 vs. 13%) and Saccharides (6 vs. 0%). It is interesting in the latter category that the only occasion when GCFs of this biosynthetic class were identified was in SUK42. We also examined BIG-SCAPE networks found in different strains (Supplementary Figure S2) and revealed that GCF FAM_00285 (Region 8.8, Supplementary Table S2) was found in all strains, including *S. albus* NBRC 13014 (Supplementary Figure S2B). This GCF, most accurately described in the complete genome of *K. setae* KM-6054, encodes the production of a putative NRP siderophore with 5% similarity to BGC0001593 (Kautsar et al., 2020), the ficellomycin biosynthetic gene cluster from *Streptomyces ficellus* (Liu et al., 2017). A second GCF (FAM_00311, Supplementary Figure S2B) encoding a siderophore with low similarity to the ficellomycin biosynthetic cluster was also found in Group 1 *Kitasatospora* strains except for SUK42. Members of FAM_00223 (Supplementary Figure S2B; Region 8.4, Supplementary Table S2), which encode γ-butryolactone biosynthesis (Kato et al., 2007), are also only found in Group 1 *Kitasatospora* strains, has 4% similarity to BGC0000262, the prejadomycin biosynthetic gene cluster from *Streptomyces* sp. PGA64 (Kallio et al., 2008). Two networks from the RIPP family (Supplementary Figure S2F) were shared among all Group 2 *Kitasatospora* strains, although none were found in SUK42: FAM_00111, which shares the RamAB transporters (Kodani et al., 2004) with BGC0000519, the labyrinthopeptin A2 biosynthetic gene cluster from *Actinomadura namibiensis* (Meindl et al., 2010). Despite FAM_00286 encoding the peptidase and lanthionine synthase enzymes, no propeptide was identified in this cluster by antiSMASH. Two Saccharide GCFs were found in SUK42 (FAM_00,205, Supplementary Figure S2G; Region 10.3, Supplementary Table S2 and FAM_00206, Figure 2G; Regions 11.1, Supplementary Table S2); the only member of this GCF found in the analysed strains. A member of the terpene class (FAM_00187, Supplementary Figure S2H; Region 4.4, Supplementary Table S2) was also found across the *Kitasatospora* strains; GCF, BGC0000663 (Kautsar et al., 2020) and encodes the hopene biosynthetic gene cluster first identified in *S. coelicolor* A3(2) (Bentley et al., 2002). Finally FAM_00218, (Supplementary Figure S2H; Supplementary Table S2, Region 4.5), encodes BGC0000804 (Kautsar et al., 2020), an acarviostatin I03 biosynthetic gene cluster from *Streptomyces coelicoflavus* ZG0656 in addition to a gene encoding avermitilol synthase (Guo et al., 2012).

## Discussion

Exploitation of the biodiversity of the planet will prove key to the identification of novel natural products and generate solutions to key medical and industrial challenges that are faced in the 21st century. Many areas of the world are inhabited by understudied plants; this is especially true of tropical regions such as South East Asia with the high-rainfall necessary to generate great biodiversity. This has allowed the discovery of microbial endophytes that produce a variety of new bioactive compounds (Strobel, 2003). For example, in a study of α-glucosidase inhibitory activity of extracts of plants with reported anti-diabetic activity, crude extracts of the folium of *A. neurocarpum* Miq. displayed an IC_50_ value 4.22 μg/ml for inhibition of α-glucosidase (Elya et al., 2012). In order to determine if this plant harboured any endophytes that might possess novel bioactivity, we set out to isolate a microorganism from this plant and rather, than physically screening for bioactivity, we assessed the potential for production of natural products using a genome mining approach (Blin et al., 2019). In so doing, we isolated a microorganism that we visually identified as a member of the genus *Streptomyces*, well known (Barka et al., 2016) for production of secondary metabolites. However following preliminary analysis of this isolate, we identified it as a member of *Kitasatospora* (Li et al., 2021).

This genus was first proposed in 1982, and although they are similar to members of the genus *Streptomyces*, members of *Kitasatospora* display clear differences in cell wall composition form a well-defined cluster on the basis of phylogenetic of 16S rRNA gene sequences (Takahashi, 2017). Genome sequences of 43 strains have been published so far and range in size from 6.3 to 12.36 Mb in size; these organisms were mostly isolated from soil, but others were isolated from water or insects (Li et al., 2021). The smallest genome of this genus so far sequenced, at 6.31 Mb, belongs to *Streptomyces* sp. DSM 40024 isolated under orchid grass from Japan. (Li et al., 2021). As such, the genome size of SUK42, at 6.55 Mb in size, represents the second smallest of the *Kitasatospora* genomes sequences sequenced so far and the smallest of the reference sequences (Supplementary Table S1). As this strain is the first endophytic strain of *Kitasatospora* so far sequenced, it is only through further studies of other plants and strains that will be possible to determine if a small genome size is a characteristic of other endophytic members of *Kitasatospora*.

Phylogenetic analysis of the SUK42 genome sequence in comparison with the genome sequences of other *Kitasatospora* type strains demonstrated that SUK42 likely represents a novel species and form part of clade containing *S. avellaneus*, *S. novaecaesareae*, *S. rubellomurinus,* and *K. purpeofusca*, with the former being the closest relative of SUK42 (ANI 91.80%. This clade is distinct from the other *Kitasatospora* clade generated using reference sequences alone. These two clades likely correspond the lineage II and III, respectively, recently reported for *Kitasatospora* based on all available genome sequences (Li et al., 2021). Despite its small genome, there is significant core genome shared between SUK42 and the other members of this clade. For example, SUK42 has a genome size around 2/3 that of *K. purpeofusca* and perhaps represent specialization through genomic reduction that has allowed SUK42 to adapt to its endophytic life-style. SsgB is a unique cell division protein required for the proper placement of the cytokinetic protein FtsZ in vegetative and aerial hyphae of *S. coelicolor* (Grantcharova et al., 2005). Z ring placement is a major determinant of the mycelial lifestyle of streptomycetes (Jyothikumar et al., 2008) and its conserved structure illustrates its likely similar role in *Kitasatospora*. The shared, distinctive amino acid sequence of SsgB of SUK42 and other *Kitasatospora* strains confirms SUK42 as a member of this genus (Girard et al., 2013).

The pan-genome of SUK42 and the eight *Kitasatospora* reference sequences showed that the core genome was consistent with a recent study (1,458 vs. 1,498, respectively) (Li et al., 2021). This study also demonstrated that, like *Streptomyces*, *Kitasatospora* has an open genome. On the basis of available reference sequences, it was possible to affiliate SUK42 with a clade containing the four other reference sequences Group 1 that displayed similar core genomes that differed from that of Group 2. The recent study of *Kitasatospora* genomes (Li et al., 2021) also identified four major clades within the genus and, based on the related reference sequences to SUK42, this strains lies in lineage II. It will be interesting to discover if future endophytic *Kitasatospora* genomes are found in this lineage.

Investigation of the subsystems encoded by SUK42 in comparison to the other *Kitasatospora* reference sequences revealed differences in the classes of metabolic pathways encoded by different strains. Despite its relatively small genome (∼6.55 Mb compared to an average of 8.25 Mb), SUK42 has an increased proportion of subsystems such as Cell Wall and Dormancy and a reduced proportion of subsystems like Cofactors, Iron acquisition, Virulence, Stress response and especially Nitrogen and Secondary Metabolism. It is tempting to suggest that these functions might be provided by the host plant and permitted the loss of the genetic material encoding this functionality during genome reduction of this strain. If true, this suggest that SUK42 may be undergoing streamlining selection to minimize the burden of encoding unnecessary metabolic pathways. Clearly however, the fact that we could cultivate SUK42 shows that this organism is not dependent on its host, at least in the laboratory environment. The concept that endophytes have reduced genomes as compared to their relatives and might be related to the endophytic lifestyle is not new, since genome reduction is reported in endophytic bacteria from the genus *Enterobacter* (Lopez-Fernandez et al., 2015).

The subsystem showing the largest reduction in SUK42 was the Secondary Metabolism class, so we analysed the SUK42 and *Kitasatospora* reference sequences in more details using antiSMASH and BiG-SCAPE. This analysis showed that SUK42 encoded 35 BGCs and the average for the reference sequences was 54 and illustrates that SUK42 encodes fewer BGCs than the average and is perhaps consistent with the idea that an endophyte does not need the same repertoire of bioactive weapons in its arsenal as inhabitants of the heterogeneous, complex and more competitive environment of soil. Consequently, those BCGs retained by SUK42 and found in all *Kitastaospora* reference sequences, such as FAM_00187, encoding hopene biosynthesis (Bentley et al., 2002) likely play an important role in the life-style of this organism. Intriguingly, the only members of the saccharide class of BGCs found in the *Kitasatopora* sequences were found in SUK42. A member of the Terpene GCF displayed similarity to the cluster encoding the BCG for acarviostatin I03 *from S. coelicoflavus* ZG0656 (Guo et al., 2012). This compound displays α-amylase inhibition and suggests that the inhibition first reported for the host-plant of SUK42, *A. neurocarpum* Miq (Elya et al., 2012), might be as a result of this metabolite produced by the endophyte. Glucosidase inhibitors, such as Acarviostatin, are used as treatments and prophylactics for diabetes, hyperlipoproteinemia, hyperlipidemia, obesity, or other secondary symptoms caused by these diseases (Qin et al., 2011) and highlights that clinical properties of medicinal plants may be due to compounds produced by their endophytic microbial partners. In scientific terms, intriguing questions remains about whether this terpene or the saccharides are produced *in planta* and, if so, what role they might perform. If indeed a symbiotic relationship exists between SUK42 and *A. neurocarpum*, what benefits does each partner gain from the relationship?

## Data Availability

The datasets presented in this study can be found in online repositories and the supplementary data. The names of the repository/repositories and accession number(s) can be found below: https://www.ncbi.nlm.nih.gov/, LFMD00000000.
