# Supplementary material for: Adaptation to Endophytic Lifestyle Through Genome Reduction by Kitasatospora sp. SUK42
Source: Front Bioeng Biotechnol. 2021 Oct 12;9:740722. doi: 10.3389/fbioe.2021.740722 (PMC8545861; doi:10.3389/fbioe.2021.740722)
Supplement: Supplementary file 2 [file DataSheet1.PDF]

Reduced genome of the endophyte *Kitasatospora* sp. SUK42

Noraziah M. Zin, Aisha Ismail, David R. Mark, Gareth Westrop, Jana K. Schniete and Paul R. Herron

Supplementary Tables and Figs.

Table S1. Genomic features of SUK42 and *Kitasatospora* refrence sequences used in this study. Estimated average nucleotide (ANI) was calculate dusing AutoMLST (Alanjary et al., 2019).

Table S2: BGCs encoded by SUK42. Cluster identification was carried out by antiSMASH (Blin et al., 2019) and clusters located at the edge of a contig are highlighted (*).

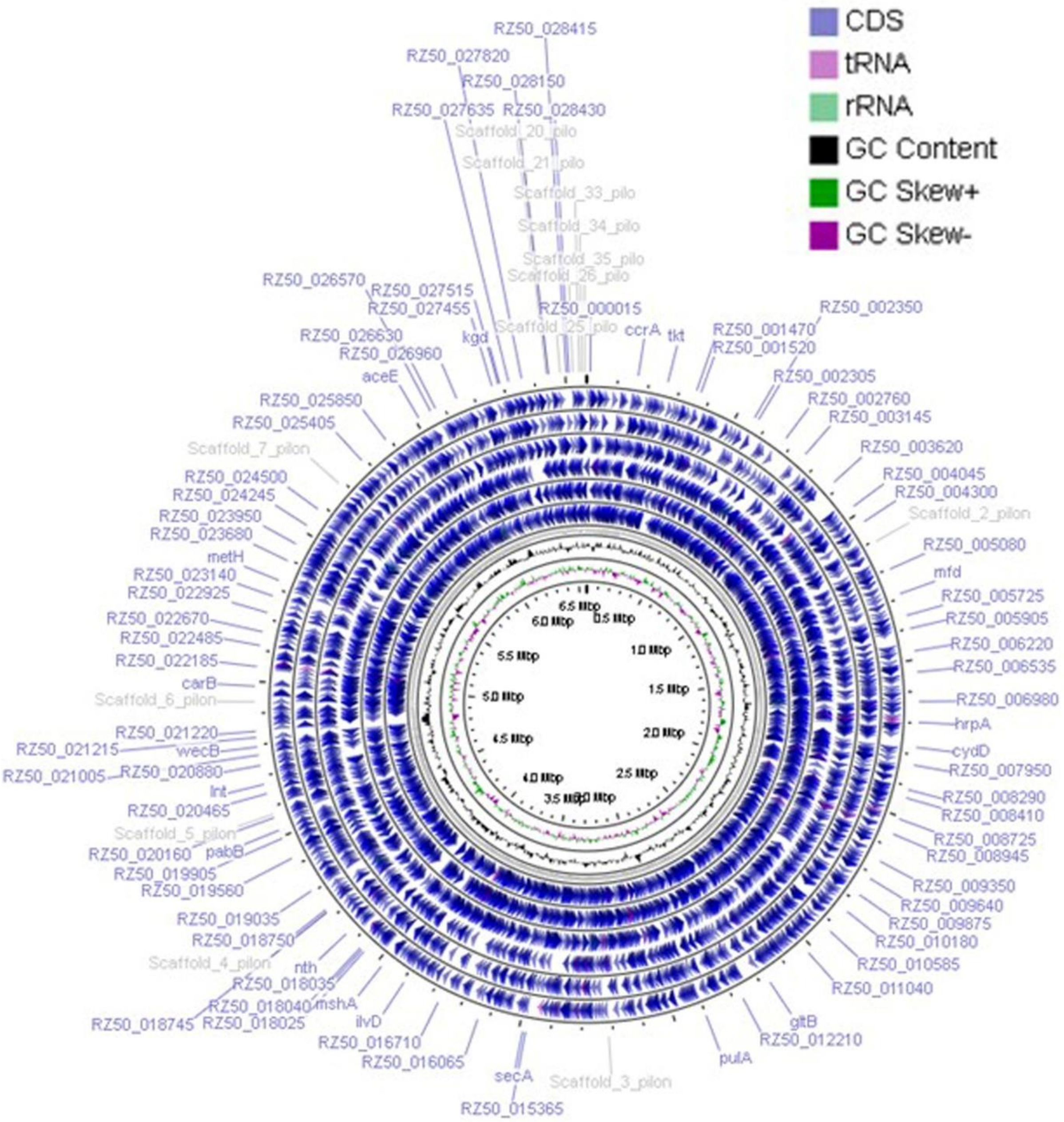

Fig. S1. Genomic map of *Kitasatospora sp*. SUK42. The genome was viewed in CGviewer (Grant and Stothard, 2008) and is represented as circles: from outside to inside, the concentric circles represent: nucleotide position; coding sequences (CDS) on the forward strand; CDS on the reverse strand; GC content (black); GC-skew on forward strand (purple); GC skew on the reverse strand (green). The genome sequence is listed in NCBI under accession number LFMD00000000 and assembly GCA_015776785.2.

Fig S2 Identification of shared gene cluster families in *Kitasatospora sp*. SUK42, other putative *Kitasatospora* reference sequences

Gene clusters that share a common genomic core, and multi-locus phylogenies of these gene clusters were determined using antiSMASH (Blin et al., 2019), BiG-SCAPE and CORASON (Navarro-Munoz et al., 2020). The CORASON outputs are displayed as newick trees, a core functional report along with the appropriate gene cluster family. Biosynthetic gene clusters belonging to the same family and shared cross several reference sequences are also displayed. (a) BiG-SCAPE Gene Cluster Family NRPS, (b) others (c), PKSI, (d) PKS-NRP_hybrids, (e) PKSother, (f) RiPPs (g) Saccharides, (h) Terpenes.

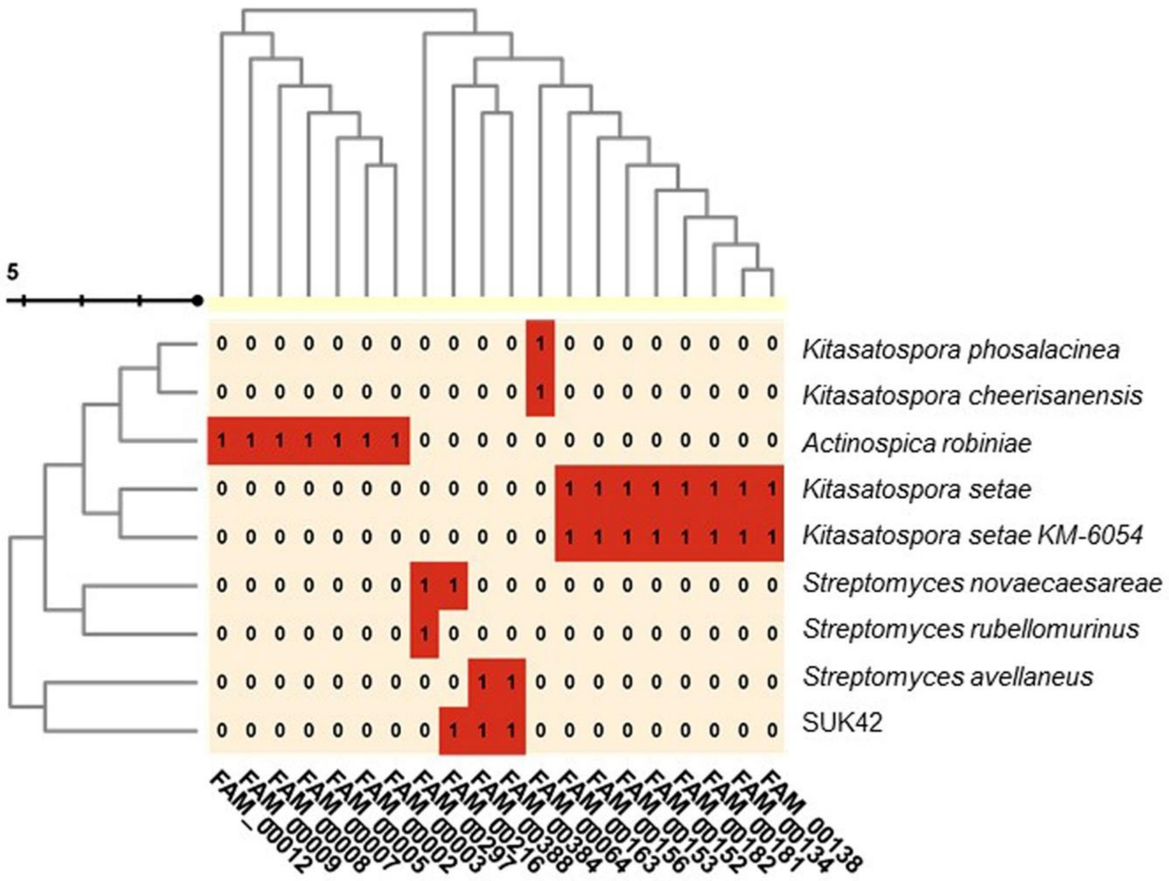
5
Kitasatospora phosalacinea
Kitasatospora cheerisanensis
Actinospica robiniae
Kitasatospora setae
Kitasatospora setae KM-6054
Streptomyces novaecaesareae
Streptomyces rubellomurinus
Streptomyces avellaneus
SUK42
FAM_00012
FAM_00009
FAM_00008
FAM_00007
FAM_00005
FAM_00002
FAM_00003
FAM_00297
FAM_00216
FAM_00388
FAM_00384
FAM_00064
FAM_00163
FAM_00156
FAM_00153
FAM_00152
FAM_00182
FAM_00181
FAM_00134
FAM_00138

Fig S2A NRPS

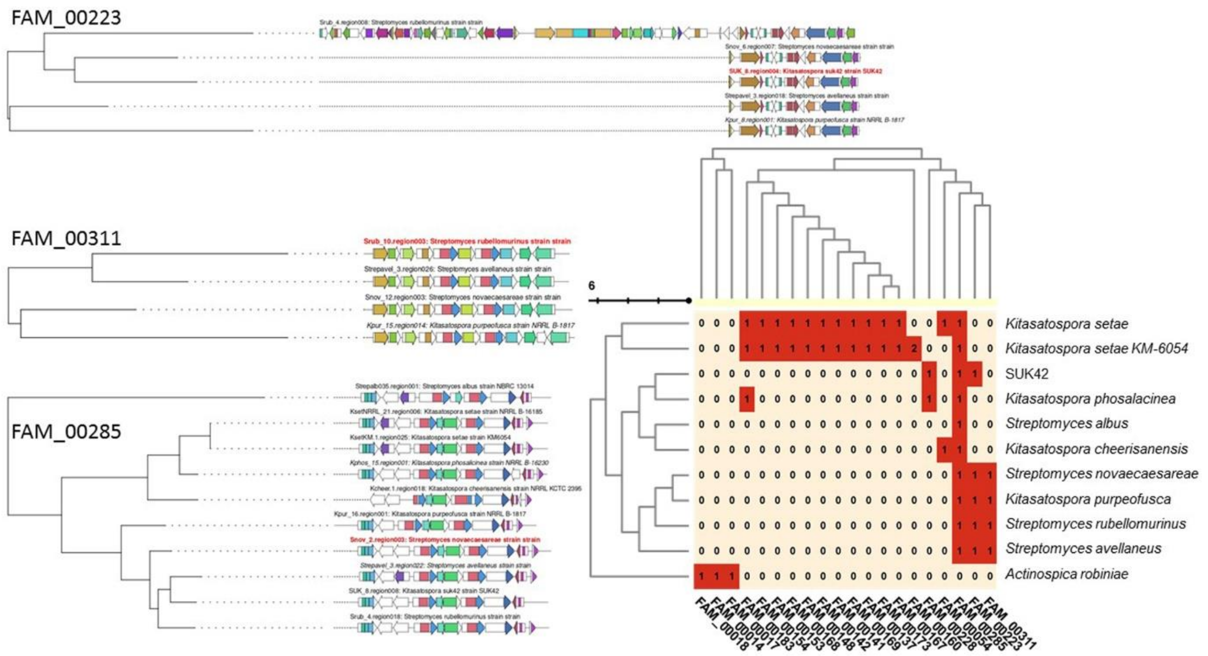

Fig S2B Others

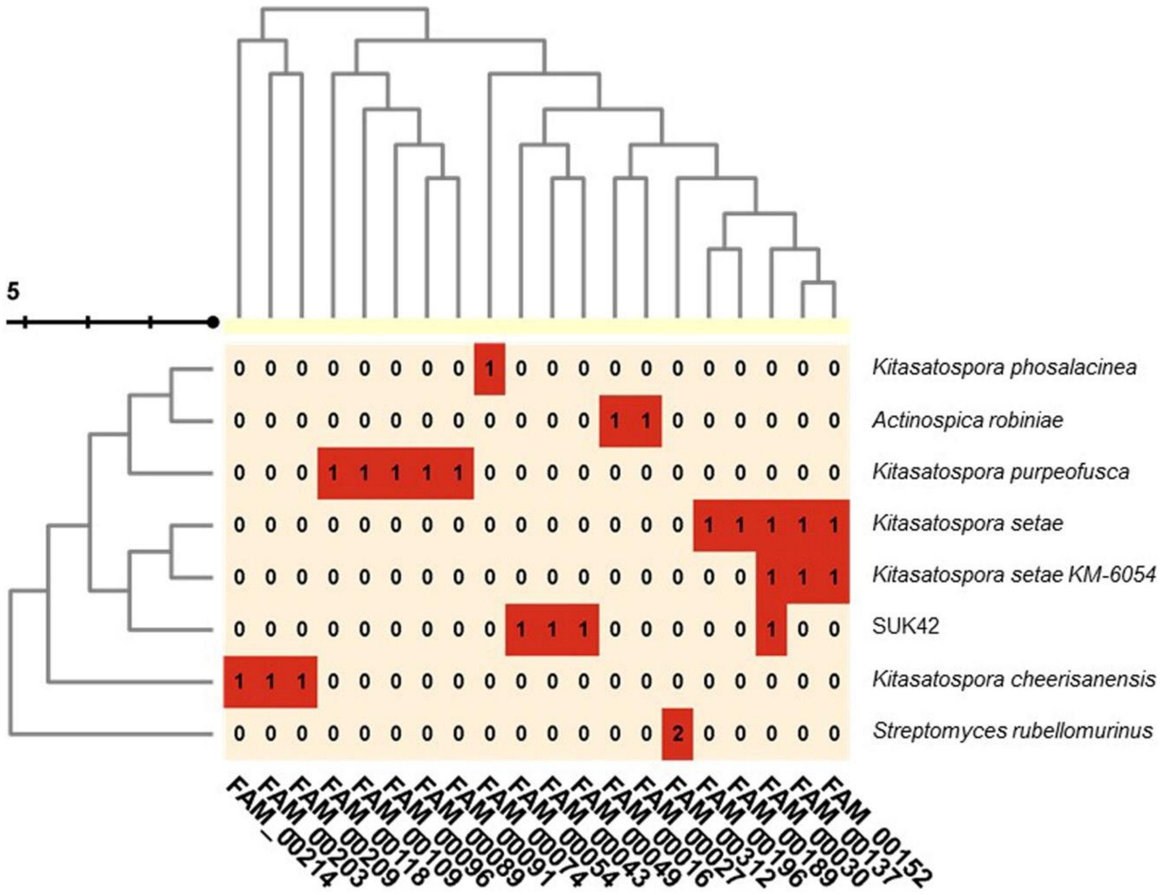
5
Kitasatospora phosalacinea
Actinospica robiniae
Kitasatospora purpeofusca
Kitasatospora setae
Kitasatospora setae KM-6054
SUK42
Kitasatospora cheerisanensis
Streptomyces rubellomurinus
FAM_00214
FAM_00203
FAM_00209
FAM_00118
FAM_00109
FAM_00096
FAM_00089
FAM_00091
FAM_00074
FAM_00054
FAM_00043
FAM_00049
FAM_00016
FAM_00027
FAM_00312
FAM_00196
FAM_00189
FAM_00030
FAM_00137
FAM_00152

FigS2C PKSI

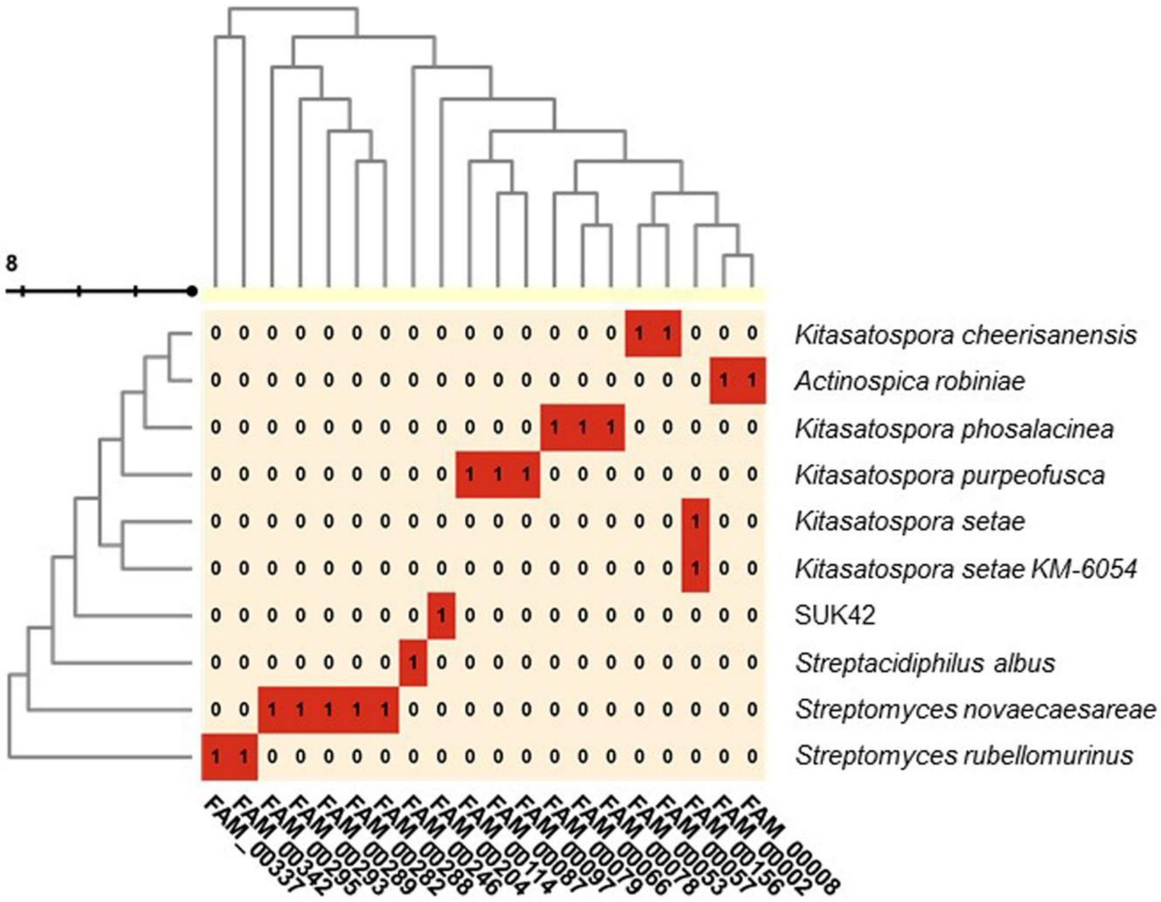

FigS2D PKS-NRP_hybrids

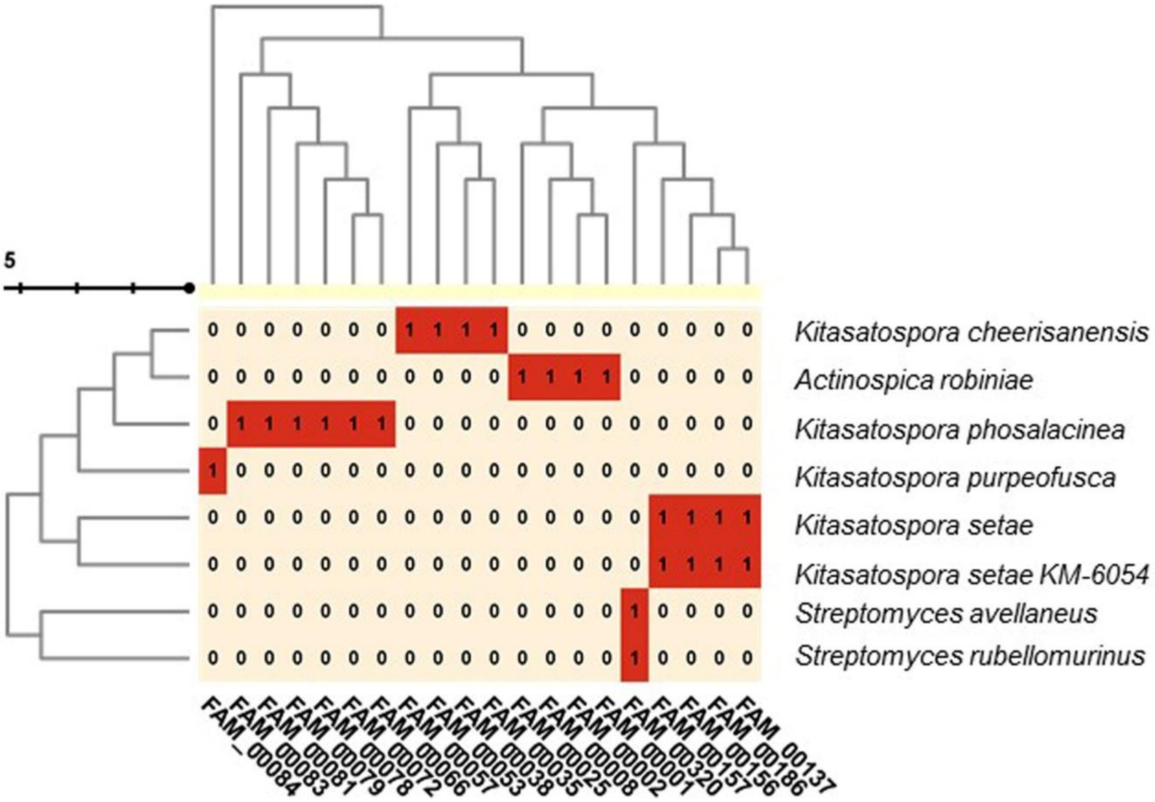

Fig S2E PKSother

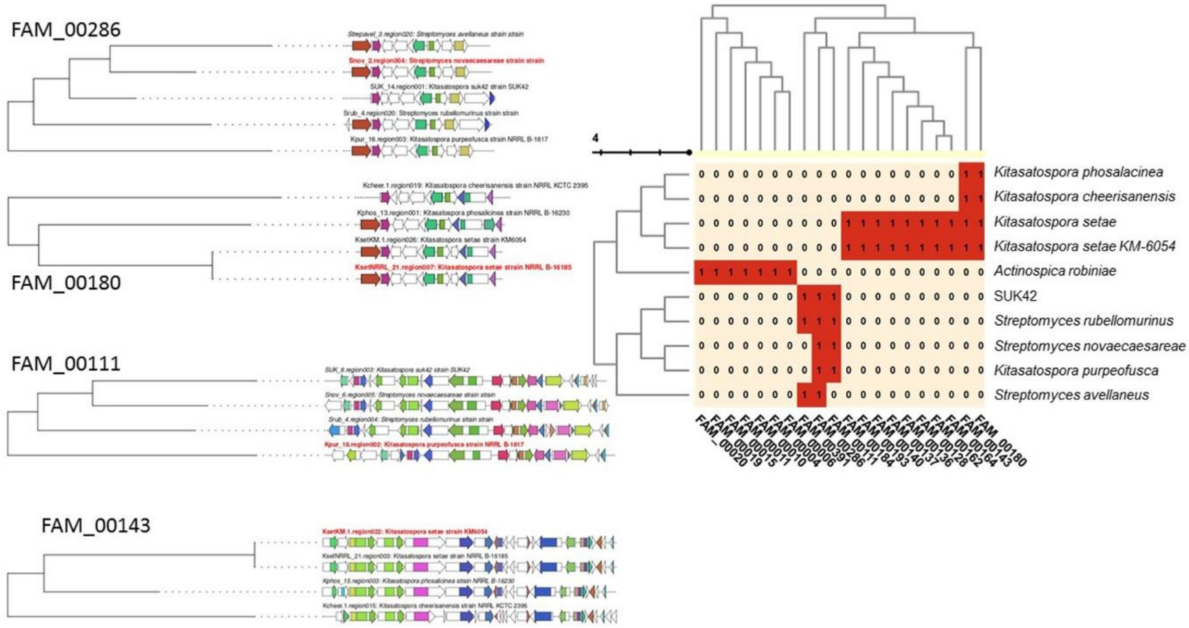

Fig S2F RiPPs

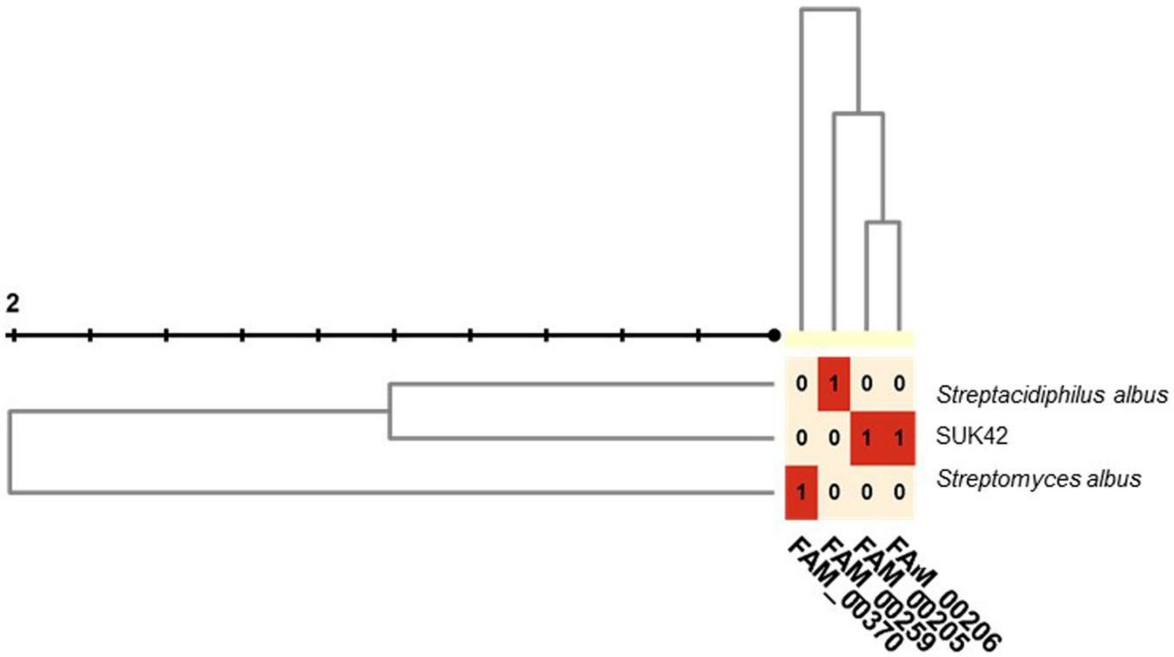

Fig S2G Saccharides

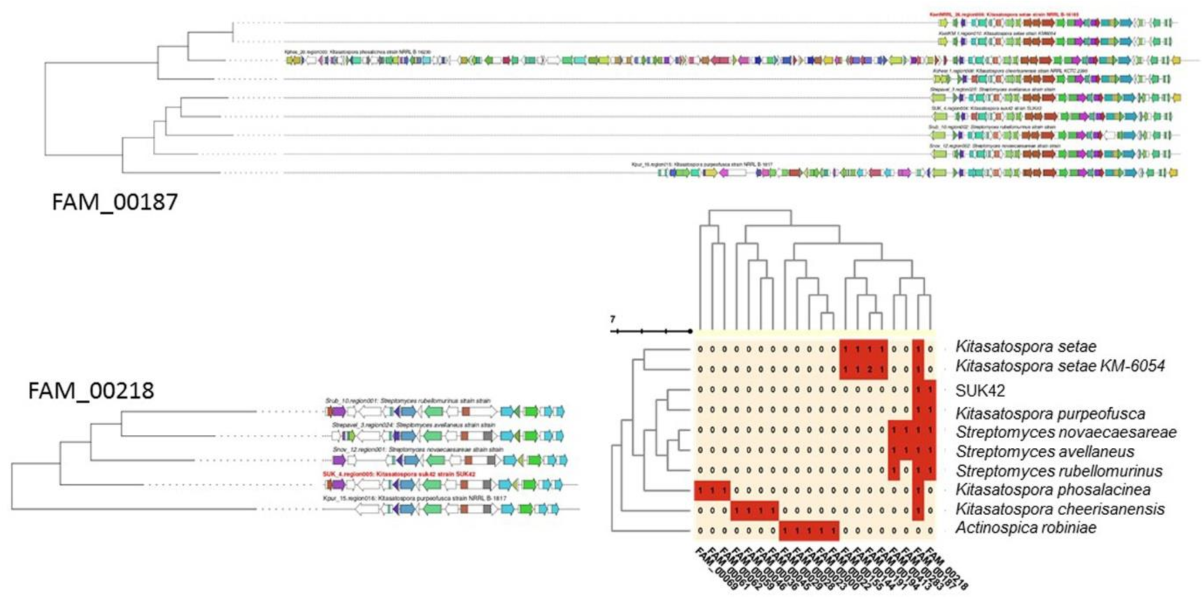

Fig S2H Terpenes.

References

ALANJARY, M., STEINKE, K. & ZIEMERT, N. 2019. AutoMLST: an automated web server for generating multi-locus species trees highlighting natural product potential. *Nucleic Acids Res,* 47**,** W276-W282.

BLIN, K., SHAW, S., STEINKE, K., VILLEBRO, R., ZIEMERT, N., LEE, S. Y., MEDEMA, M. H. & WEBER, T. 2019. antiSMASH 5.0: updates to the secondary metabolite genome mining pipeline. *Nucleic Acids Res,* 47**,** W81-W87.

GRANT, J. R. & STOTHARD, P. 2008. The CGView Server: a comparative genomics tool for circular genomes. *Nucleic Acids Res,* 36**,** W181-4.

NAVARRO-MUNOZ, J. C., SELEM-MOJICA, N., MULLOWNEY, M. W., KAUTSAR, S. A., TRYON, J. H., PARKINSON, E. I., DE LOS SANTOS, E. L. C., YEONG, M., CRUZ-MORALES, P., ABUBUCKER, S., ROETERS, A., LOKHORST, W., FERNANDEZ-GUERRA, A., CAPPELINI, L. T. D., GOERING, A. W., THOMSON, R. J., METCALF, W. W., KELLEHER, N. L., BARONA-GOMEZ, F. & MEDEMA, M. H. 2020. A computational framework to explore large-scale biosynthetic diversity. *Nat Chem Biol,* 16**,** 60-68.
